# Bioguided Fractionation Shows *Cassia alata* Extract to Inhibit *Staphylococcus epidermidis* and *Pseudomonas aeruginosa* Growth and Biofilm Formation

**DOI:** 10.1155/2012/867103

**Published:** 2012-04-02

**Authors:** Samuel Takashi Saito, Danielle da Silva Trentin, Alexandre José Macedo, Cristina Pungartnik, Grace Gosmann, Jaqueline de Deos Silveira, Temenouga Nikolova Guecheva, João Antonio Pêgas Henriques, Martin Brendel

**Affiliations:** ^1^Laboratório de Biologia de Fungos, Centro de Biotecnologia e Genética, Universidade Estadual de Santa Cruz, Ilhéus, BA 45662-900, Brazil; ^2^Faculdade de Farmácia, Universidade Federal do Rio Grande do Sul, Porto Alegre, RS 90610-00, Brazil; ^3^Centro de Biotecnologia e Departamento de Biofísica, Universidade Federal do Rio Grande do Sul, Porto Alegre, RS 91501-970, Brazil; ^4^Genotox Royal, Universidade Federal do Rio Grande do Sul, Porto Alegre, RS 91501-970, Brazil; ^5^Instituto de Biotecnologia, Universidade de Caxias do Sul, Caxias do Sul, RS 95070-560, Brazil

## Abstract

Plant extracts have a long history to be used in folk medicine. *Cassia alata* extracts are known to exert antibacterial activity but details on compounds and mechanism of action remain poorly explored. We purified and concentrated the aqueous leaf extract of *C. alata* by reverse phase-solid phase extraction and screened the resulting CaRP extract for antimicrobial activity. CaRP extract exhibited antimicrobial activity for *Pseudomonas aeruginosa*, *Staphylococcus epidermidis*, *S. aureus*, and *Bacillus subtilis*. CaRP also inhibited biofilm formation of *S. epidermidis* and *P. aeruginosa*. Several bacterial growth-inhibiting compounds were detected when CaRP extract was fractionated by TLC chromatography coupled to bioautography agar overlay technique. HPLC chromatography of CaRP extract yielded 20 subfractions that were tested by bioautography for antimicrobial activity against *S. aureus* and *S. epidermidis*. Five bioactive fractions were detected and chemically characterized, using high-resolution mass spectrometry (qTOF-MS/MS). Six compounds from four fractions could be characterized as kaempferol, kaempferol-O-diglucoside, kaempferol-O-glucoside, quercetin-O-glucoside, rhein, and danthron. In the *Salmonella*/microsome assay CaRP showed weak mutagenicity (MI < 3) only in strain TA98, pointing to a frameshift mutation activity. These results indicate that *C. alata* leaf extract contains a minimum of 7 compounds with antimicrobial activity and that these together or as single substance are active in preventing formation of bacterial biofilm, indicating potential for therapeutic applications.

## 1. Introduction

*Cassia alata* L. (also known as *Senna alata*) is a shrub belonging to the *Fabaceae* family, found in intertropical areas. It is commonly known as candle bush, with reference to the shape of its inflorescences. It is annual or biannual shrub with an offensive smell, 1–4 m tall, preferring sunny and moist areas [1, 2]. Ethnopharmacological data available in a recent review [1] describe various medical applications of *C. alata* by populations from Africa (Uganda, Ghana, and Nigeria), Indonesia, and Latin America (Guatemala, Martinique, and Brazil). Leaves, flowers, and fruits of *C. alata *are used as antidiabetic, anti-inflammatory, analgesic, against digestive problems, and infectious diseases (as antibacterial and antifungal agents) [2–5]. Amongst the secondary metabolites are steroids, flavonoids, anthraquinones, anthrones, and a few less common compounds such as ellagitannin, naphthalene, phenolic acid, purine, and xanthone [1, 6–8]. Of special interest are compounds such as kaempferol glycosides and anthraquinones, already proven to have antimicrobial properties [1, 8].

The widespread use of *C. alata* in folk medicine [1] has stimulated several scientific studies to search for its pharmaceutically relevant compounds. Biological investigations regarding anti-infection properties of *C. alata* extracts demonstrate action against Gram-positive bacilli (*Bacillus subtilis*) and cocci (*Enterococcus faecalis*, *Staphylococcus aureus*, and *S. epidermidis*) as well as Gram-negative bacilli such as *Escherichia coli*, *Proteus vulgaris,* and *Pseudomonas aeruginosa* [9]. Extracts were also found to act against *Aspergillus niger*, *Candida albicans*, *Cryptococcus neoformans,* and other fungi [2, 3].

Nonetheless, the ability of *C. alata* extracts to prevent bacterial adhesion and biofilm formation remains poorly explored. In this respect, biofilms are defined as a consortium of microorganisms that are attached to a biotic or abiotic surface [10]. Compared with their planktonic counterparts, microbial cells living in biofilms have extraordinary resistance to the immune defense responses of the host as well as to biocides and antimicrobial agents [11]. They have also been shown to colonize a wide variety of medical devices and to be associated with several human diseases [12], with *S. epidermidis* and *P. aeruginosa* being the most prevalent pathogens involved in these infections [11, 12].

Solid-phase extraction (SPE) has been successfully used to obtain root extract of *C. alata* prior to the use of High Performance Liquid Chromatography (HPLC) [6]. However, SPE has still not been applied to obtain *C. alata *leaf extracts with the main goal of trace substance enrichment, matrix simplification, and medium exchange in analytical applications such as in the bioautography agar overlay (BAO) method [13, 14]. BAO is an alternative and more efficient version to the commonly used disc diffusion method in the screening of natural compounds with antimicrobial potential as it allows direct activity testing after planar chromatography of extracts [14]. The BAO method allows evaluation of the compound via its diffusion from a Thin Layer Chromatography (TLC) plate into an overlay film of agar medium containing the microorganism to be tested for susceptibility. This methodology has some advantages as it (i) can be used for bioassay-guided isolation of compounds and (ii) simplifies the identification and isolation of an active compound from the crude extract [14].

In this study we therefore combined the SPE and BAO methods to analyze the traditionally used decoction of *C. alata *for compounds with antimicrobial activities, especially those preventing bacterial growth and biofilm formation. Also, we investigated the extracts' ability to interact with DNA by performing genotoxicity assays with the crude extract to evaluate its safety using a prokaryotic model.

## 2. Materials and Methods

### 2.1. Microorganisms and Culture Conditions

Microorganisms tested in this study were *Staphylococcus aureus* (ATCC25921), *Staphylococcus epidermidis* (ATCC35984), *Pseudomonas aeruginosa* (ATCC27853), *Escherichia coli* (ATCC25923), *Bacillus subtilis*, *Salmonella choleraesuis*, *Klebsiella pneumonia*, *Saccharomyces cerevisiae,* and *Candida albicans* (CCMB286). Bacteria were obtained from the American Type Culture Collection (Manassas, USA) and clinical isolates from volunteers of the Hospital Itabuna/BA-Brazil. *Salmonella typhimurium *TA97a, TA98, TA100, TA102, and TA1535 were kindly provided by B. M. Ames (University of California, Berkeley, CA, USA). *S. cerevisiae* yeast strains were obtained from EUROSCARF (Frankfurt, Germany) and *C. albicans* from the Coleção de Culturas de Microrganismos da Bahia, UEFS (Feira de Santana-BA, Brazil). All bacterial strains were grown overnight at 37°C in Mueller-Hinton Broth (Merck) before tests and yeast strains were grown in liquid YPD (yeast extract 1%, peptone 2% and dextrose 2%) for 2-3 days at 30°C in a rotatory shaker (New Brunswick, G76) to attain stationary growth phase.

### 2.2. Solvents and Reagents

Acetonitrile (MeCN) HPLC-grade was purchased from Tedia (USA). Water was purified on a Milli-Q system (Millipore, USA). Ethanol (p.a.) was obtained from Merck (Germany) while ethyl acetate (p.a.), methanol (p.a.), and acetone (p.a.) was obtained from F. Maia (Brazil). Streptomycin, chloramphenicol, ciprofloxacin, and trifluoroacetic acid (TFA) for spectroscopy were purchased from Sigma-Aldrich (USA). All culture media were purchased from Merck (Germany) and Oxoid (England).

### 2.3. Plant Material

Leaves of *C. alata *were collected at Fazenda Ibaiti and at the campus of Universidade Estadual de Santa Cruz, in Salobrinho, Ilhéus, BA, Brazil in August 2010. The lyophilized plant material was stored at room temperature in desiccators in the dark. Voucher specimen were deposited at UESC herbarium and identified by Professor Luiz Alberto Mattos (curator) and also maintained at our laboratory for future reference (Accession on August 12, 2010).

### 2.4. Extraction Procedure

#### 2.4.1. Aqueous Extraction to Obtain CaAE

Aqueous extracts (CaAE) were prepared by decoction using lyophilized leaves [1 : 20; (w : v)] (80°C, 30 min) followed by filtration, rotary evaporation, and freeze-drying. One hundred grams of milled leaves yielded 25.4 ± 2.9% (percentage extract of dry weight) of crude aqueous extract. Lyophilized CaAE was stored in a freezer at −20°C until use.

#### 2.4.2. Reverse Phase-Solid Phase Extraction to Obtain CaRP

*Cassia alata *fraction (CaRP) was obtained using solid phase extraction (SPE) methodology. SPE was performed using the column Strata C18E 5 g/20 mL Giga Tubes (Phenomenex, USA). One g of CaAE was diluted in 100 mL of distilled water. The cartridge was conditioned with acetone (25 mL) and washed with 5 mL of water before loading the sample (100 mL). It was then washed with 20 mL of water and eluted with 25 mL of ethyl acetate. After evaporation this eluent yielded the CaRP fraction. One gram of CaAE yielded 6.4 ± 1.4% of CaRP that was stored in desiccators in the dark at room temperature until further use. The values represent mean and standard deviation of at least 3 individual extractions.

### 2.5. Thin Layer Chromatography (TLC)

One mg of each sample was dissolved in either 2 mL of water (CaAE) or 2 mL of methanol (CaRP) and 40 *μ*L (final concentration of 20 *μ*g/spot) was submitted to TLC on silica gel G60 F254 aluminum plates (Merck, 10 cm × 10 cm). Eluent was ether/ethyl acetate/formic acid (75 : 25 : 1), according to Wagner and Bladt [15]. Spots were detected by UV light at 254 and 365 nm.

### 2.6. Bioautography Agar Overlay (BAO)

Bioautography using TLC plates plays an important role in the search for active compounds from plants, giving quick access to information concerning both the activity and the localization of the activity in complex plant matrices. TLC chromatograms were placed into sterile Petri dishes (20 cm *∅*) and sterilized using UVC (254 nm) light for 15 min before being covered by an overlay of bacterial growth media (40 mL Mueller-Hinton agar (Oxoid, England) containing 0.1% Triton-X) or yeast media (YPD). After agar solidification 400 *μ*L of microbial suspension (2-3 × 10^8^ cells/mL) was spread onto the surface and then incubated for either 24 h (bacteria) or 48 h (yeast). During this growth period compounds from the TLC plate could diffuse into agar and exert possible cytostatic or cytotoxic action on the growing microorganisms. After the incubation agar plates were sprayed with 2 mg/mL (w/v) aqueous solution of *p*-iodonitrotetrazolium (INT) (Sigma-Aldrich, USA) and incubated for 2–6 h. Microbial growth led to the emergence of purple-red color resulting from the reduction of INT to formazan. Clear zones in the agar indicated the presence of compounds that had inhibited microbial growth [16]. Ciprofloxacin, streptomycin, and chloramphenicol were used as positive controls. Results and photo represent one of at least 3 independent experiments.

### 2.7. Biofilm Quantification of CaRP

*S. epidermidis *ATCC35984 and *P. aeruginosa* ATCC27853 were used as models of bacterial biofilm formation. A bacterial suspension (3 × 10^8^ CFU/mL) in 0.9% NaCl was used in the assays. A protocol adapted from Antunes and coworkers [17], using crystal violet in 96-well flat bottom microtiter plates (Costar 3599, Corning, USA), was applied. Distinct concentrations (0.125 to 20.0 mg/mL) of CaRP in ethanol were tested. Two hundred *μ*L of each concentration was aseptically evaporated at room temperature during 7 h, so that 0.025 to 4.0 mg of CaRP remained in each well. Wells from evaporated pure ethanol were used as control since they allowed 100% of biofilm formation. After ethanol evaporation each well received 100 *μ*L of bacterial suspension plus 100 *μ*L of the tryptone soya broth (Oxoid Ltd., England) and the plates were incubated at 37°C for 24 h. The suspension was then removed and the wells washed twice with sterile saline. The remaining attached bacteria (the biofilm) were heat-fixed at 60°C for 1 h and stained with crystal violet for 15 min at room temperature. After removing excess stain, the cell-bound crystal violet was solubilized with DMSO (Sigma-Aldrich, USA) and its absorbance was measured at 570 nm (Spectramax M2e Multimode Microplate Reader, Molecular Devices, USA). Values higher than 100% (extract-free control) represent a stimulation of biofilm formation in comparison to the control. Planktonic bacterial growth was monitored by calculating the difference between the OD_600_ absorbance measured at the end and at the beginning of the incubation time. Results represent media and standard deviation of at least 3 independent experiments.

### 2.8. Scanning Electron Microscopy

Biofilms of *S. epidermidis* ATCC35984 and of *P. aeruginosa* ATCC27853 were grown in 96-well microtiter plates as described above, with a piece of Permanox sterile cell culture slide (Nalge, Nunc International, USA) added. After 24 h of incubation at 37°C, the slide samples were withdrawn from the cultures and fixed in 2.5% glutaraldehyde for 4 h, washed with 100 mM cacodylate buffer (pH 7.2), and dehydrated in increasing concentrations of acetone, according to Trentin and coworkers [18]. The Permanox slides were dried by the CO_2_ critical point technique (CPD 030 Balzers, Liechtenstein), fixed on aluminum stubs, covered with gold film, and examined in a JEOL JSM-6060 scanning electron microscope.

### 2.9. Fluorescence Microscopy of Bacterial Biofilm Cells

For fluorescence microscopy, cells were grown in the presence or absence of CaRP as described in Section 2.7 and suspended according to Stepanovic and collaborators [19] with slight adaptations. Briefly, 100 *μ*L of cell suspension washed twice with saline by centrifugation in a microcentrifuge. Cells/biofilm were resuspended in 25 *μ*L of saline by vortexing and submitted to 20 min of ultrasound pulses for biofilm disruption. Then, propidium iodide (PI) was added (1 : 1 v/v) to a final concentration of 2 *μ*M, incubated at room temperature in the dark for 30 min. PI-labeled cells were washed twice with 400 *μ*L of saline and then observed under fluorescence microscope DMRA2 (Leica) attached with PI filters. Images were captured using ×40 objectives under bright field as well as under fluorescent filters using the IM50 software (Leica).

### 2.10. LC-UV Microfractionation of CaRP

Micro-fractionation of CaRP was performed according to Queiroz and collaborators [20], with slight modifications. Reversed-phase HPLC of CaRP fraction was performed on a Shimadzu LC-20AT Prominence with detector UV-Vis SPD-20A and Collector FRC-10A (Japan). The separations were achieved on a Gemini C-18 semipreparative column Phenomenex (150 × 10 mm I.D.; 5 *μ*m 110 Å) with MeCN-water (5 : 95 to 95 : 5; 20 h). Sample injection volume was set at 250 *μ*L (10 mg), and flow-rate was 0.2 mL/min; the UV traces were measured at 254 and 347 nm. Twenty fractions of 4 mL were collected in plastic tubes for every peak with level >80,000 *μ*V and named F1 to F20 (Figure 3). The content of each tube was concentrated and resuspended in 60 *μ*L of MeOH. Fifty *μ*L of each fraction was used for bioautography and mass spectroscopy (MS) analysis. 

### 2.11. ESI-qTOF-MS

Each dried fraction (F_6_, F_11_, F_13_, F_18_, and F_20_) obtained from LC-UV-fractionation was submitted to direct infusion in an ESI-q-TOF mass spectrometer (Waters Q-TOF mass spectrometer). The data was obtained in mass spectrometer Q-TOF micro from Micromass and processed using the MassLynx V4.1 software package. High-purity nitrogen was used as nebulizer and auxiliary gas. Argon was used as collision gas. ESI collision energy (CE) was betwen 4 and 45 V for negative ion mode. Desolvation temperature was set at 350°C and source temperature was set at 120°C. The desolvation and cone gas flows were 350 L/h and 70 L/h, respectively. The sample cone voltage was set at 33 V, the extraction voltage was set at 2.5 V, and the capillary voltage was set at 2.5 kV. The mass scan range was from 50 to 1500 *m/z*.

### 2.12. Salmonella/Microsome Assay

Mutagenicity was assayed by the preincubation procedure. The S9 metabolic activation mixture (S9 mix) was prepared according to Maron and Ames [21]. Briefly, 100 *μ*L of test bacterial cultures (1-2×10^9^ cells/mL) were incubated at 37°C with different amounts of CaRP in the presence or absence of S9 mix for 20 min, without shaking. Subsequently, 2 mL of soft agar (0.6% agar, 0.5% NaCl, 50 *μ*M histidine, 50 *μ*M biotin, pH 7.4, 42°C) were added to the test tube and poured immediately onto a plate of minimal agar (1.5% agar, Vogel-Bonner E medium, containing 2% glucose). Aflatoxin B1 (1 *μ*g/plate) was used as positive control for all strains in the presence of metabolic activation (with S9 mix). In the absence of metabolic activation, 4-nitroquinoline-oxide (4-NQO, 0.5 *μ*g/plate) was used for TA97a, TA98, and TA102 strains, and sodium azide (1 *μ*g/plate) for TA100 and TA1535 strains. Plates were incubated in the dark at 37°C for 48 h before counting the revertant colonies. A test substance was considered mutagenic when significant dose response and ANOVA variance were observed, and the increase in the mean number of revertants on test plates was at least twofold higher than that observed in the negative control plates (or MI ≥ 3 for TA1535 strain).

### 2.13. Statistical Analysis

Data were calculated as the mean ± standard deviation of at least 3 independent experiments. ANOVA (Dunnet's test) was used for the statistical analysis (*P* < 0.05). For AMES test, the results were analyzed by the *Salmonella *Statistic Assay (Environmental Monitoring System Laboratory, EPA-Software Version 2.3, April 1988).

## 3. Results

### 3.1. Bioautography

Our first goal was to screen CaAE and its fraction CaRP against bacteria of medical interest, using the TLC-BAO (Table 1). Results demonstrated that CaAE did not inhibit any of the tested microorganisms, while CaRP presented antibacterial activity against *S. aureus*, *S. epidermidis*, *B. subtilis* and *P. aeruginosa*.

### 3.2. Growth Inhibition

**Figure 1:**
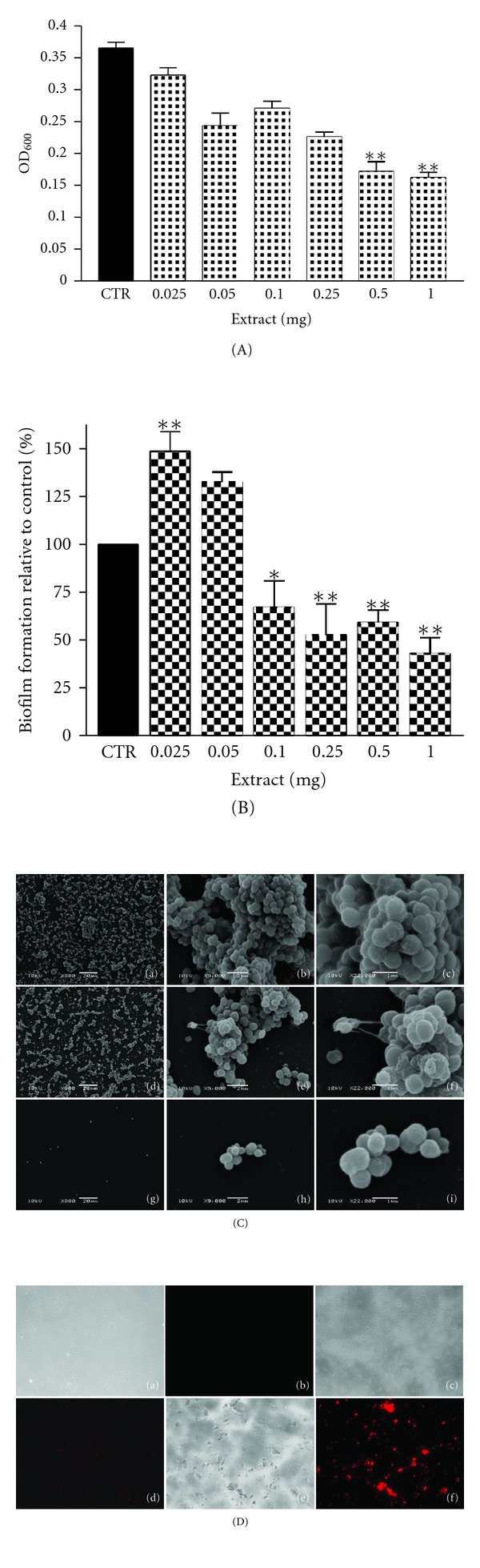
(A) Growth of CaRP-treated *S. epidermidis *ATCC35984, CRT: control; (B) inhibition of biofilm formation of *S. epidermidis*  *P < 0.5 and **P < 0.01 related to control (100%); (C) Scanning electron microscopy of *S. epidermidis* treated with CaRP (a) to (c) control; (d) to (f) 0.05 mg; (g) to (i) 0.5 mg of extract; (D) fluorescence microscopy: (a-b) at inoculation; (c-d) 0.05 mg; (e-f) 0.5 mg of extract mature biofilm. Note: (D)-f cells are clumped.

**Figure 2:**
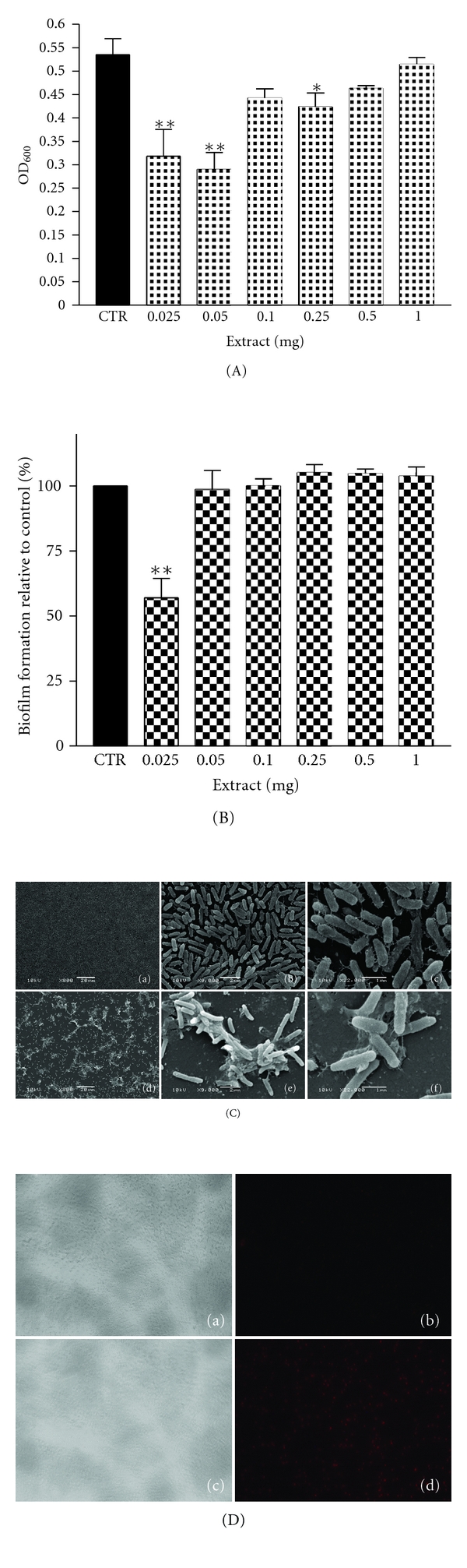
(A) Growth of CaRP-treated *P. aeruginosa *ATCC27853, CRT: control; (B) Inhibition of biofilm formation of *P. aeruginosa*  *P < 0.5 and **P < 0.01 related to control (100%); (C) scanning electron microscopy of *P. aeruginosa *treated with CaRP (a) to (c) control; (d) to (f) 0.025 mg of extract. *P < 0.5 and **P < 0.01 related to control (100%); (D) fluorescence microscopy: (a-b) at inoculation; (c-d) 0.025 mg of extract, mature biofilm.

Bacterial growth inhibition of *S. epidermidis *by CaRP at doses from 0.025 to 1 mg was dose dependent, being significant at doses of 0.5 mg or higher (*P* < 0.01) when compared to the control (Figure 1(A)). There was no clear dose dependence in survival when measuring growth inhibition of *P. aeruginosa. *Although CaRP fraction could inhibit the growth at the lowest doses (0.025 to 0.25 mg) it showed no effect at 1.0 mg (Figure 2(A)).

### 3.3. Biofilm Formation Assay

We tested the ability of CaRP to prevent biofilm formation of *S. epidermidis* and *P. aeruginosa* (Figures 1(B) and 2(B)) at doses from 0.025 to 1.0 mg. CaRP inhibited biofilm formation of *S. epidermidis* at doses from 0.1 to 1.0 mg when compared to control in a dose-dependent manner (Figure 1(B)). Regarding *P. aeruginosa* biofilm formation, CaRP could inhibit biofilm formation up to 50% only at the lowest dose (0.025 mg), while it had no influence at all other doses (Figure 2(B)).

### 3.4. Scanning Electron Microscopy and Fluorescence Microscopy

The effect of CaRP fraction upon *S. epidermidis *and* P*. *aeruginosa *biofilm morphology was evaluated by scanning electron microscopy (SEM) (Figures 1(C) and 2(C)). SEM of CaRP-treated cells confirmed the crystal violet data (Figure 1(B)), showing that *S. epidermidis *biofilm formation occurred at 0.05 mg and not at 0.5 mg (Figure 1(C)). Cells in the control were clearly attached to the substratum, forming bacterial clusters (Figure 1(C)-(a to c)). In treated biofilms, at 0.05 mg, a high number of cell clusters was observed (Figure 1(C)-(d to f)), while at 0.5 mg of CaRP the number of attached bacterial cells was lower and they appeared only in small clusters or even as single cells (Figure 1(C)-(g to i)). Considering *P. aeruginosa*, images show that the number of bacterial aggregates decreased when compared with the control (Figure 2(C)-(a to c)); however an overproduction of EPS matrix was observed at 0.025 mg (Figure 2(C)-(d to f)). Fluorescence microscopy using propidium iodide showed that almost all treated cells present in the formed biofilm structure were dead, equally for *S. epidermidis* (Figure 1(D)-(c to f)) or for *P. aeruginosa* (Figure 2(D)-(c to d)) when compared to untreated cells (at inoculation) (Figure 1(D)-(a and b), and Figure 2(D)-(a and b), resp.). Once again we could observe that the number of biofilm cells was reduced by the treatments (mature biofilm) and that control cells were viable, since they were visible in bright field but were not stained by propidium iodide.

### 3.5. LC-Microfractionation and Bioautography

**Figure 3:**
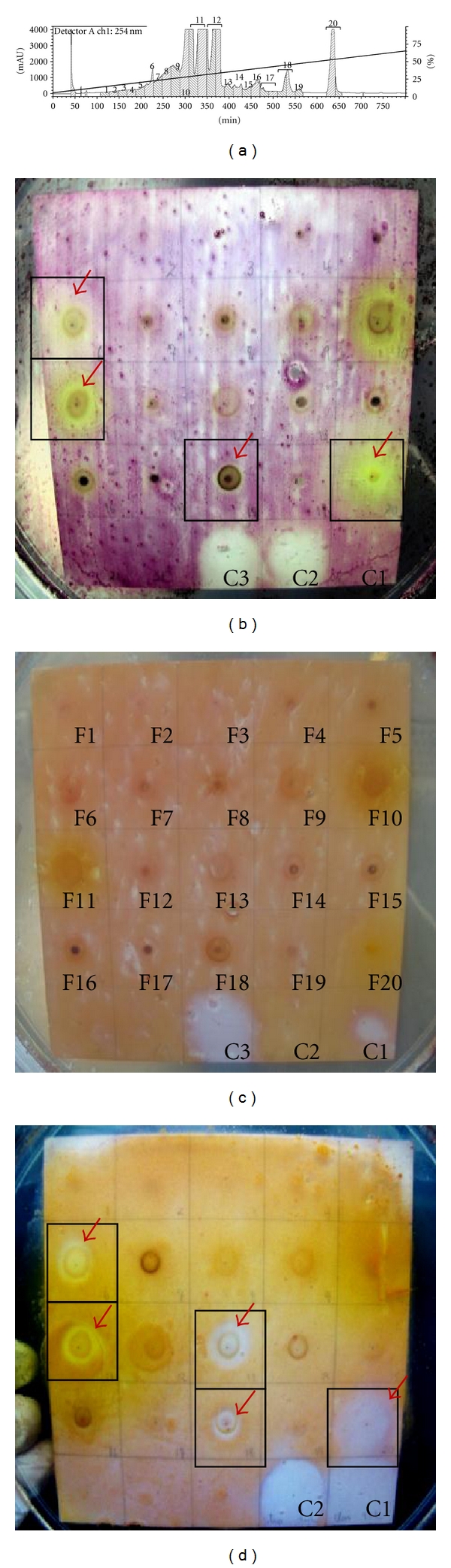
CaRP LC microfractionation and test for bacteriocidic action of spotted fractions via BOD TLC. (a) LC chromatogram revealing CaRP fractions; bioautography of each fraction against (b) *Staphylococcus epidermidis*; (c) *Pseudomonas aeruginosa*; against* Staphylococcus aureus*. Controls marked as C1 (Streptomycin 26 *μ*g), C2 (Chloramphenicol 24 *μ*g), and C3 (Ciprofloxacin 6 *μ*g).

Since CaRP was shown to be active against some bacteria, it was microfractioned by LC in a semipreparative RP-column (Figure 3). Twenty fractions were obtained and further spotted on TLC plates and tested for antibacterial activity against the *S. epidermidis*,* S. aureus,* and* P. aeruginosa* (Figure 3(b) to Figure 3(d)). From these fractions, five presented antibacterial activity: F_6_ [yield of 2.3%], F_11 _[52.6%], F_13_ [3.9%], F_18_ [1.5%], and F_20_ [1.2%]. Fractions F_6_, F_11_, F_18_, and F_20_ inhibited growth of *S. aureus* (Figure 3(c)) and *S. epidermidis *(Figure 3(d)) whereas fraction F_13_ was only active against *S. aureus *(Figure 3(c)). None of the fractions in the concentrations (F_1_ to F_20_) tested showed activity against *P. aeruginosa *(Figure 3(d)). The controls revealed *P. aeruginosa *as chloramphenicol resistant (Figure 3(b), C2) and only sensitive to ciprofloxacin and streptomycin (Figure 3(b), C3 and C1); *S. epidermidis* was streptomycin resistant (Figure 3(d), C1) and sensitive to ciprofloxacin and chloramphenicol while *S. aureus* was sensitive to streptomycin and chloramphenicol (Figure 3(c), C1 and C2).

### 3.6. Compound Identification in CaRP

Kaempferol-O-diglucoside, quercetin-O-glucoside, kaempferol-O-glucoside, kaempferol, rhein, and danthron were identified from high-resolution qTOF-MS/MS data as the major active compounds in fractions F_6_, F_11_, F_18_, and F_20_ obtained from CaRP after LC microfractionation (Table 3). It was not possible to identify the compounds present in F_13_.

### 3.7. Salmonella/Microsome Mutagenicity Assay

Results on genotoxicity are shown in Table 2. The CaRP dose range was determined in a range finder experiment in strain TA100, with and without metabolization, and cytotoxicity was not observed at concentrations up to 5000 *μ*g/plate (data not shown). In the mutagenicity assay the dose range between 1000 and 5000 *μ*g/plate was used. The mutagenic effect of the extract on TA98 strain (detects frameshift mutation in the DNA target –C–G–C–G–C–G–C–G) in the absence of metabolic activation indicated that some components of the extract could effectively interact with DNA. However, the effect of the extract on the frameshift mutation-detecting strain TA97a (detects frameshift mutations in –C–C–C–C–C–C–; +1 cytosine) was not significant. Also, no mutagenicity was seen in the strains detecting base pair substitutions in the absence or presence of metabolic activation: TA1535 and the corresponding isogenic strain TA100 (both detect base pair substitutions of a leucine-coding GAG triplet to a proline-coding GGG). Negative results were also observed in strain TA102, which is sensitive to oxidative and alkylating mutagens (detects transversions or transitions in TAA DNA sequences).

## 4. Discussion

*Cassia alata *had been mainly used in folk medicine against constipation and skin diseases [1], and recently biotechnological applications of *C. alata* extracts have been proposed for cosmetic industry [1, 22]. The effectiveness of *C. alata* aqueous extract against *S. aureus*, *S. pyogenes*, *E. coli*, *P. vulgaris*, *P. aeruginosa,* and *C. albicans* has been reported using decoction and MIC [4, 9].

We performed a bioguided-activity fractionation of an aqueous extract of *C. alata *(CaAE) employing a cleaner SPE extraction to obtain the CaRP extract, and a faster and more economic method, combining TLC and BAO to detect antimicrobial activity. We also investigated the extract's influence on bacterial biofilm formation of *S. epidermidis* and *P. aeruginosa*. Although the crude extract (CaAE) did not show antimicrobial activity using TLC-BAO (Table 1), CaRP presented antibacterial activity against *S. aureus*, *S. epidermidis, B. subtilis, and P. aeruginosa*, but was inactive against *E. coli*, *C. albicans, Salmonella choleraesius*, *Klebsiella pneumonia,* and *Saccharomyces cerevisiae* demonstrating that the fractionation was successful. The efficiency and validity of TLC-BAO method could be verified with positive controls of antibiotics (Table 1).

Pathogenic biofilm-forming microorganisms are focus of intensive research due to their involvement in a large number of chronic infectious diseases and medical device-related infections [12]. This indicates the need to search for new antimicrobial resources, including plants used in traditional medicine that may contain a great variety of compounds with therapeutic properties [18].

Inhibition of bacterial growth and of biofilm formation by CaRP was dose dependent (Figure 1(A), 1(B) and 1(C)) in *S. epidermidis*. SEM images showed that CaRP prevented significantly formation of biofilm at the highest dose used (Figure 1(B) and 1(C)). Cells in the control were clearly cemented to the substratum and formed nascent cell cluster (Figure 1(C)-(a to c)) while the amount of cells in the clusters, embedded in the EPS matrix was diminished after CaRP treatment (Figure 1(C)-(d to i)). It seems that bacterial growth was inhibited before the cells were able to promote adhesion on the surface (Figures 1(A) and 1(C)). In the same way, it was recently shown via SEM that *S. epidermidis* exhibited different morphology after treatment with vancomycin, that is, there was a differential impact on *S. epidermidis *morphology in the center and periphery of biofilm upon treatment, suggesting a spatial distribution of vancomycin-induced damage in *S. epidermidis* biofilm [23]. Regarding *P. aeruginosa* there was no dose dependence effect, neither in growth inhibition (Figure 2(A)) nor in antibiofilm activity according to the results of the OD_600_ measurements and CV method, respectively (Figure 2(B)). Interestingly, the bacterial growth was only inhibited significantly at low dose of CaRP (Figure 2(A)) and CaRP inhibited 43% of biofilm formation only at 0.025 mg (Figure 2(B)). Although the number of bacterial aggregates decreased, an overproduction of EPS matrix was observed (Figure 2(C)-(d to f)). The matrix production in *P. aeruginosa* is regulated by the quorum sensing (QS) system. QS is a bacterial cell-cell communication which associates specific genes' transcription with cell density [24]. Since matrix production is modulated by CaRP it is therefore plausible that some kind of aberrant regulation of QS occurs in this process. Analyzing the data together, it seems that CaRP probably triggers the modulation of EPS production in *P. aeruginosa* and, therefore, the cell organization (Figure 2(C)-(b and e)). The recovery of the ability to form biofilm by *P. aeruginosa* at higher doses of CaRP might be related to some fraction component(s), which compensate the observed antibiofilm activity at low doses. Results obtained by fluorescence microscopy corroborate with the growth inhibition data, proving that the antibiofilm CaRP activity is closely related to growth inhibition, as almost all cells present in the biofilm structure are dead (Figures 1(D) and 2(D)).

Since CaRP demonstrated different antibacterial effects, we expected that it might contain distinct active compounds. Microfractionation and TLC (Figure 3) showed 4 subfractions active against *S. epidermidis *but none of them was active against *P. aeruginosa* (Figures 3(b) and 3(c)). This may be explained by the small amount of substance in each fraction that did not any longer allow inhibitory activity on a bacterium with higher resistance. The alternative explanation for the observed antibacterial activity of CaRP could be a synergistic interaction of at least 2 compounds of this fraction. In order to establish a general antibacterial activity of some fractions we used the same method against *S. aureus* (Figure 3(d)). Indeed, the same 4 fractions (F_6_, F_11_, F_18_, and F_20_) plus one (F_13_) promoted growth inhibition in this bacterium.

In order to identify the secondary metabolites of *C. alata* that could be responsible for cytotoxicity and biofilm inhibition, we performed high-resolution mass spectrometry of the obtained subfractions from CaRP. It was possible to identify four flavonoids (present in F_6_, F_11_, and F_18_) and two anthraquinones (present in F_20_) (Table 3). Flavonoids have been described as health-promoting, disease-preventing dietary supplements, and to have activity as cancer-preventive agents. Additionally, they are considered extremely safe and have low toxicity, making them excellent candidates for chemopreventive agents [25]. Flavonoids, such as kaempferol and quercetin are known to have a wide range of pharmacological activities, including antioxidant, anti-inflammatory, antimicrobial, anticancer, cardioprotective, neuroprotective, antidiabetic, antiosteoporotic, estrogenic/antiestrogenic, anxiolytic, analgesic, and antiallergic activities [26]. However, it still remains to be determined whether these properties, for example, those of quercetin, are affected independently or share a common mechanism of action [27]. Natural compounds are reported to inhibit biofilm formation by various mechanisms without affecting the microbial growth rate [28, 29]. It is possible that the various known flavonoids may have differential modes of action in inhibiting formation of biofilm. Indeed, both kaempferol and quercetin have recently been shown to be effective antagonists of cell-cell signaling and to suppress biofilm formation in *Vibrio harveyi* and *E. coli* O157:H7 cultures (at doses ranging from 6–100 *μ*g/mL), indicating a potential modulation by these compounds of bacterial cell to cell communication. Similarly, both molecules in our study could act synergistically in their contribution to inhibit biofilm formation of *S. epidermidis *and* P. aeruginosa *(Figures 1(C) and 2(C)).

While flavonoids are usually considered beneficial for human health [25], anthraquinones such as danthron (1,8-dihydroxyanthraquinone) have been described as possible human carcinogens [30] and to induce DNA damage and apoptosis in various mammalian cells at a dose range of 25–100 *μ*g/mL [31]. They still have not yet been described as responsible for biofilm inhibition. Identification and quantification of anthraquinones in biological matrices have been widely described and this improved considerably our understanding of their mechanism of action. However, their biological targets have not yet been totally defined [32]. Anthraquinones are chemical derivatives of quinones, which are believed to react with sulfhydryl (–SH) groups, a critical reaction since blocking of –SH groups of enzymes may inhibit their activity. Oxidative reaction with –SH groups will also change the cellular redox potential [33]. Inhibition of the catalytic activity of topoisomerase II has been shown to contribute to anthraquinone-induced genotoxicity and mutagenicity [34].

One possible mechanism of action of danthron is that it may cause DNA damage particularly at guanines in the presence of Cu(II), cytochrome P450 reductase, and the NADPH-generating system, which led to the conclusion that oxidative DNA damage by danthron may be relevant for the initiation of cancer [35].

We have observed that growth inhibition of *S. epidermidis* by CaRP was dose dependent (Figure 1(A)), while that of *P. aeruginosa* (inhibition only at lowest doses) was not. Since it is known that inhibition of biofilm formation may occur without affecting growth rate [28, 29], this could be interpreted that anthraquinones rather than flavonoids may negatively interact with DNA or enzymes, and that biofilm inhibition may be, therefore, a consequence of both biologically toxic events.

Regarding the fact that folk medicine uses whole plant extracts as remedy we also assayed the whole extract (CaRP) for its genotoxicity. Our data on mutagenicity show CaRP to be slightly mutagenic, but only in strain TA98 (Table 2). Recently, danthron has been shown to be mutagenic in *S. typhimurium* strain TA102 in presence of exogenous metabolic activation (S9 mix) and weakly mutagenic in TA1537 with or without metabolic activation [31, 36] while rhein was found to be mutagenic in tester strain TA102 [37]. A review of the data related to the safety of quercetin reports lack of evidence of *in vivo* toxicity, including lack of genotoxic/carcinogenic properties [27], while data regarding the safety of kaempferol is conflicting: some studies show kaempferol to induce antimutagenic activity [26], while other reports have revealed that this flavonoid may induce genotoxic effects [26]. With respect of our data of weak genotoxicity of CaRP in the Salmonella/microsome assay, we may speculate that anthraquinones rather than flavonoids are the causal agents.

## 5. Conclusions

As important conclusions of our work we may summarize our conclusion as follows:

- This is the first study showing the ability of *C. alata* metabolites upon two important biofilm-forming pathogens.
- The antibiofilm CaRP activity is closely related to growth inhibition, as almost all cells present in the treated biofilm structure are dead.
- This is the first study presenting the genotoxicity evaluation for this very common and much-used medicinal plant.
- Our extract characterization identified the major components of CaRP as 4 flavonoids and 2 anthraquinones.
- *C. alata* might be a source of compounds that inhibit biofilm formation.

## Figures and Tables

**Table 1:** Antimicrobial activity of *C. alata* leaf extract by TLC-BAO.

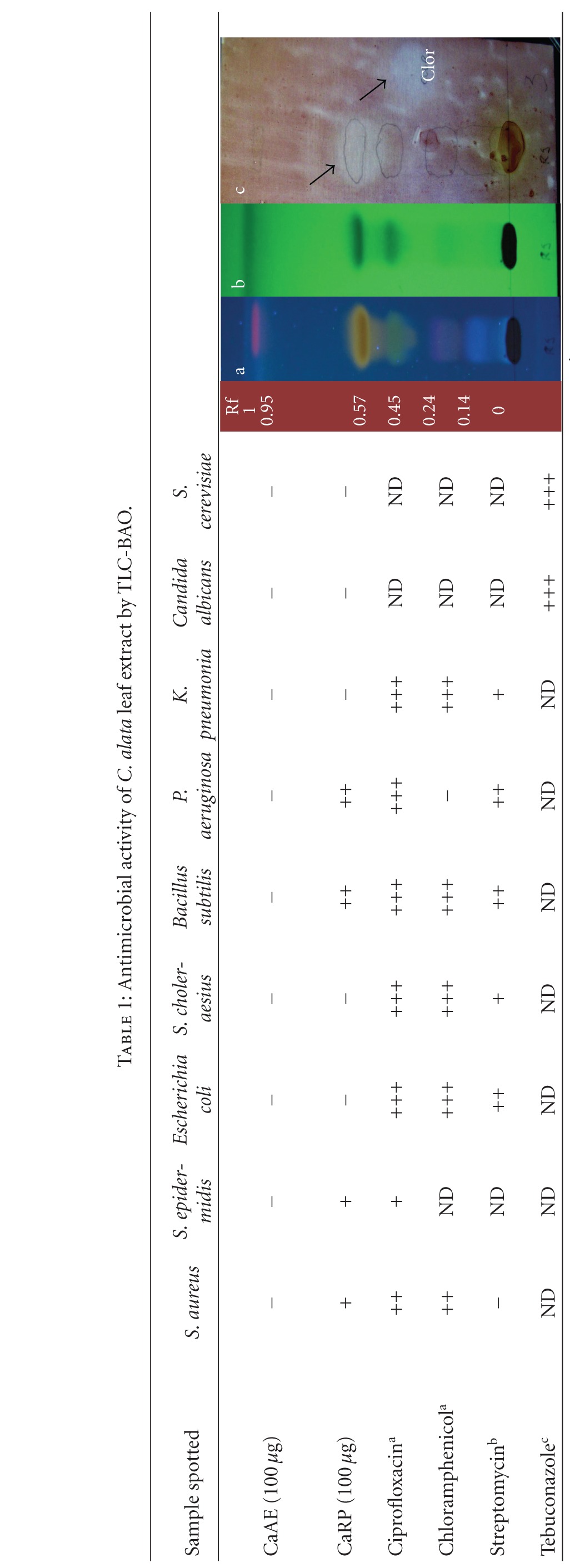

+: <0.5 cm of diameter of inhibition; ++: <1.0 cm of diameter of inhibition; +++: >1.0 cm of diameter of inhibition; −: resistant cells; ND: not determined; ^a^18 *μ*g; ^b^19 *μ*g; ^c^50 *μ*g.

TLC-BAO representative figure; a: TLC exposed to UV_254 nm_, b: TLC exposed to UV_350 nm_; c: Bioautography of *S. epidermidis*. Clor: chloramphenicol 18 *μ*g. Rf: retention factor. Arrows indicate nongrowing cells.

**Table 2:** High-resolution mass spectrometric data of major secondary metabolites identified in the HPLC fractions by qTOF-MS/MS.

Chemical class	Fraction	Molecular weight	MS^1^ (*m/z) *	MS^2^ (*m/z*)^a^	Identified compound
Flavonoids	F6	594.15847	593.1835 [M-H]^−^	593.1453, 429.0766, **284.0342,** 255.0322	Kaempferol-O-diglucoside
	F6	464.095476	463.1127 [M-H]^−^	463.0873, 301.0361, **300.0290**, 255.0326, 179.0036	Quercetin-O-glucoside
	F11	448.100561	447.1187 [M-H]^−^	447.1440, **284.0699**, 255.0648, 227.0681, 151.0299	Kaempferol-O-glucoside
	F18	286.047738	285.0687 [M-H]^−^	**285.0738**, 229.0806, 185.0870, 171.0709	Kaempferol

Anthraquinones	F20	284.032088	283.0597 [M-H]^−^	283.2830, 257.0650, **239.0532**, 211.0573, 183.0626	Rhein
	F20	240.042259	239.0664 [M-H]^−^	239.0538, 211.0580, **183.0630**	Danthron

^a^Ions in bold face indicate the most intense product ion (100% relative intensity). Compounds present in F13 were not identified.

**Table 3:** Induction of *his*+ revertants in *S. typhimurium *strains by CaRP with and without metabolic activation (S9 mix).

*S. typhimurium*strains											
Substance	Concentration (*μ*g/plate)	TA98		TA97a		TA100		TA1535		TA102	
		Rev/plate^a^	MI^b^	Rev/plate^a^	MI^b^	Rev/plate^a^	MI^b^	Rev/plate^a^	MI^b^	Rev/plate^a^	MI^b^
Without metabolic activation (−S9)											

NC^c^	—	15.3 ± 1.5	—	86.3 ± 15.8	—	123.0 ± 13.1	—	8.3 ± 3.2	—	258.0 ± 40.1	—
CaRP	1000	24.3 ± 4.7	1.59	103.0 ± 20.2	1.19	137.0 ± 19.0	1.11	7.6 ± 2.0	0.92	237.0 ± 15.5	0.92
	2000	25.0 ± 2.6*	1.63	88.0 ± 11.7	1.02	123.7 ± 13.2	1.01	20.3 ± 12.8	1.44	260.0 ± 9.5	1.01
	3000	28.0 ± 9.6	1.83	117.6 ± 31.0	1.36	127.0 ± 8.6	1.03	12.0 ± 4.2	1.44	249.6 ± 12.4	0.97
	4000	29.6 ± 0.5**	1.94	126.6 ± 21.3	1.47	125.7 ± 21.3	1.02	9.3 ± 2.5	1.12	253.3 ± 23.5	0.98
	5000	42.3 ± 3.0**	**2.76**	111.0 ± 5.5	1.29	141.0 ± 6.1	1.15	14.3 ± 2.5	1.72	297.6 ± 11.5	1.15
PC^d^	0.5 (4NQO) 1 (NaN_3_)	156.3 ± 3.8**	**10.2**	492.0 ± 62.9**	**5.7**	469.7 ± 67.3**	**3.82**	401.3 ± 26.8**	**48.2**	1485.7 ± 186.2**	**5.76**

With metabolic activation (+S9)											

NC^c^	—	58.3 ± 8.0	—	106.6 ± 6.8	—	119.0 ± 15.9	—	10.6 ± 3.5	—	325.6 ± 8.3	—
CaRP	1000	68.0 ± 9.5	1.17	109.3 ± 7.5	1.02	115.0 ± 9.5	0.97	12.3 ± 4.6	1.15	318.6 ± 15.9	0.98
	2000	71.3 ± 1.5	1.22	130.3 ± 17.0	1.22	110.0 ± 7.2	0.92	9.0 ± 2.6	0.84	384.6 ± 21.2*	1.18
	3000	68.3 ± 5.5	1.17	136.0 ± 12.1	1.28	120.0 ± 1.7	1.01	13.0 ± 5.0	1.22	335.6 ± 25.7	1.03
	4000	60.6 ± 8.5	1.04	131.6 ± 9.2*	1.24	121.0 ± 10.4	1.02	10.3 ± 3.0	0.97	394.0 ± 23.0*	1.21
	5000	64.0 ± 7.5	1.10	132.6 ± 11.0	1.24	149.3 ± 21.2	1.25	13.0 ± 3.6	1.22	338.6 ± 28.5	1.04
PC^d^	1 (AFB_1_)	393.3 ± 98.2**	**6.74**	466.7 ± 32.3**	**4.38**	362.7 ± 136.6**	**3.05**	172.0 ± 57.2**	16.12	854.7 ± 134.6**	**2.62**

^a^Number of revertants/plate: mean of three independent experiments ± SD; ^b^MI: mutagenic index (no. of *his*+ induced in the sample/no. of spontaneous *his*+ in the negative control); ^c^PC: positive control (−S9) sodium azide to TA100 and TA1535; 4-NQO to TA97a, TA98 and TA102; (+S9) aflatoxin B1 for all strains; ^d^NC: negative control distillated water (10 *μ*L) used as a solvent for the extract. *Data significant in relation to the negative control *P* < 0.05; **P < 0.01.
